# Long term outcomes following critical care hospital admission: A prospective cohort study of UK biobank participants^✰,★^

**DOI:** 10.1016/j.lanepe.2021.100121

**Published:** 2021-07

**Authors:** Joanne McPeake, Theodore J Iwashyna, Philip Henderson, Alastair H Leyland, Daniel Mackay, Tara Quasim, Matthew Walters, Michael Harhay, Martin Shaw

**Affiliations:** a Intensive Care Unit, Glasgow Royal Infirmary, Glasgow, United Kingdom; b MRC/CSO Social and Public Health Sciences Unit, University of Glasgow, Glasgow, United Kingdom; c Centre for Clinical Management Research, VA Ann Arbor Health System, Ann Arbor, MI, United States of America; d Department of Internal Medicine, Division of Pulmonary and Critical Care, University of Michigan, Ann Arbor, Michigan, United States of America; e School of Medicine, Dentistry and Nursing, University of Glasgow, Glasgow, United Kingdom; f Institute of Health and Wellbeing, University of Glasgow, Glasgow, United Kingdom; g Department of Biostatistics, Epidemiology, and Informatics; and Palliative and Advanced Illness Research (PAIR) Center, Perelman School of Medicine, University of Pennsylvania, Pennsylvania, United States; h Clinical Physics, NHS Greater Glasgow and Clyde, Glasgow, United Kingdom

**Keywords:** Recovery, Long-term outcomes, Intensive care and emotional

## Abstract

**Background:**

: This study aimed to understand the impact of a critical care admission on long-term outcomes, compared to other hospitalised patients without a critical care encounter. A secondary aim was to examine the interrelationship between emotional, physical, and social problems during recovery.

**Methods:**

: We utilised data from the UK Biobank, an on-going, prospective population-based cohort study. We employed propensity score matching to assess differences in outcomes between patients with a critical care encounter and patients admitted to the hospital (first admission to hospital available) without critical care. Structural equation modelling was used to analyse emotional, physical and social outcomes following critical illness and the relationships between these health domains.

**Findings:**

: Data from 1,618 patients were analysed. The median time to follow-up in the critical care cohort was 4427 days (IQR:788–6146) vs 4516 days (IQR: 811–6369) in the non-critical care, hospitalised cohort. Across the two time periods assessed (pre and post 2000), patients exposed to critical care were more likely to experience mental health issues such as depression (*p* < 0.01) and social isolation (*p* = 0.01) following discharge from hospital. The critical care cohort were also more likely to have social problems such as the requirement for government funded welfare support (*p* = 0.02). In the critical care cohort, social and emotional health were closely correlated (*p* < 0.001, 95% CI:0.33–0.54). The nature of physical problems changed over time; pre-2000 there was a significant difference between the critical and non-critical care in physical outcomes following discharge from hospital, however, there was no difference detected between the two cohorts post-2000.

**Interpretation:**

This cohort study has demonstrated that survivors of critical illness have different psycho-social outcomes to matched patients, hospitalised without a critical care encounter.

**Funding:**

JM is funded by a THIS.Institute (University of Cambridge) Research Fellowship (PD-2019–02–16). AHL is part of the Social and Public Health Sciences Unit, funded by the Medical Research Council (MC_UU_12017/13) and the Scottish Government Chief Scientist Office (SPHSU13).

**Research in context**

**Evidence before**

Following critical illness, many patients experience long term physical, social, emotional and cognitive issues. At present, there is limited evidence demonstrating the benefit of any specific intervention.  To support intervention development in this area, more evidence is required into the unique features of survivorship and how often complex problems, influence multiple aspects of recovery.

**Added value**

Using data from over 1600 UK Biobank participants, we assessed differences in outcomes between patients with a critical care encounter and patients admitted to the hospital without critical care.  Uniquely, we explored any differences in key economic outcomes, such as the use of welfare benefits.  Structural equation modelling was used to analyse how different aspects of recovery (physical, emotional and social problems) were related to one another.

**Implications of all available evidence**

This study found a significant difference in emotional and social outcomes for critical care and non-critical care hospitalised participants, after matching for individual demographics, comorbidities and nature of hospitalisation.  Further, it has shown that physical, social and emotional problems are interrelated.  Alongside previous evidence, this study has highlighted that future interventional research, aimed at improving outcomes for survivors of critical illness, must address all aspects of recovery if measurable, meaningful improvements to patient care are to be achieved.

Alt-text: Unlabelled box

## 1 Introduction

Patients who have been admitted to an Intensive Care Unit (ICU) are known to be at risk for long-term problems [1], [2]. These encompass physical, emotional, cognitive and social issues and have been termed Post Intensive Care Syndrome (PICS) [3], [4], [5], [6].

In response, clinicians have sought to provide targeted therapies to improve outcomes, including rehabilitation programmes and informal support programmes [7], [8], [9]. At present, there is limited randomised trial evidence demonstrating the benefit of any specific intervention [10]. This has led to increased debate about whether PICS is indeed a unique feature of ICU survivorship or, the poor long-term outcomes seen are merely a feature of pre-existing or deteriorating chronic conditions present before ICU admission [11], [12].

The existing work in this area has important gaps. First, often a single outcome is the focus, limiting the ability to understand the interrelationships amongst various features of recovery. Secondly, optimal comparator cohorts are often lacking. Third, samples are often drawn from a single centre or after a substantial selection process (such as enrolment in a randomised clinical trial). Finally, the research question is often framed as a false dichotomy, rather than seeking to quantify the extent of possible influence, recognizing the complexity of multiple aspects of recovery.

Therefore, we sought to advance the literature by using data from the UK Biobank to ask: (a) what is the interrelationship between emotional, physical, and social problems amongst patients following a critical care admission? (b) to what extent is care in critical care associated with worse recovery? We focus on survivors, as it is well known that ICU patients face worse short-term mortality.

## 2 Methods

### 2.1 Data and patients

The UK Biobank is a prospective population-based cohort study established to support the study of lifestyle, environmental and genetic determinants of adulthood diseases [13]. Between 2006 and 2010, the Biobank recruited over 500,000 participants aged between 40–70 from the general population; the response rate was 5.5%. Those participants enroled and attended 1 of 22 assessment centres across the UK, where they completed a variety of questionnaires and had physical measures obtained [14]. The UK Biobank continues to follow up this cohort of patients and intermittently undertakes further assessment, in-person and online. The UK Biobank study was approved by the North West Multicentre Ethics Research Committee; participants provided written informed consent. This study is part of UK Biobank project 57617 (NHS National Research Ethics Service Ref: 11/NW/0382). The dataset for this analysis was accessed and generated in April 2020. Patients who withdrew consent from the UK Biobank following this date, were removed from the analysis.

## 3 Participants

### 3.1 Study cohorts

**Fig. 1:**
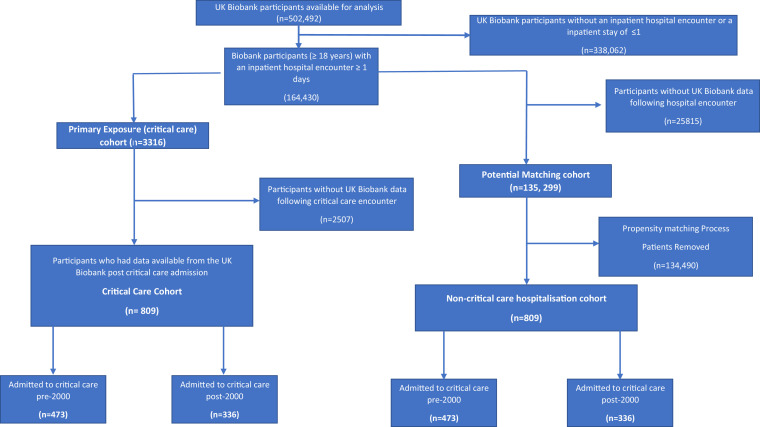
Flow of participants and match of patients with a critical care encounter.

We identified two study cohorts from the UK Biobank (Fig. 1). The primary cohort were those with a critical care admission, who had outcome data available from a Biobank assessment centre visit, following admission. This cohort was defined by Consultant Speciality (critical or intensive care) within the UK Biobank dataset (**S1**). We considered the first critical care and first hospital admission for the comparison cohorts, to accurately reflect baseline status. We utilised data from the immediate proceeding assessment centre visit after the first critical care admission. All participants in the UK Biobank, who were admitted to critical care and had assessment centre data available after their critical care admission, were included in this analysis.

The second cohort was a group of hospitalised patients, with similar baseline characteristics, not admitted to critical care. Only patients who had been admitted to hospital for a day or more were included.

### 3.2 Demographics

Area-level socioeconomic deprivation was assessed by the Townsend Index, operationalised at the output area (with approximately 300 people in each area in 2011) in which the respondent's home postcode was recorded [15]. Comorbidities were classified using the Elixhauser Co-morbidity Index and the Charlson Co-morbidity Index [16], [17]. The Elixhauser Index represents a set of 30 comorbidity measures and is used widely in health service research [18], [19]. Both indices were utilised to allow for the inclusion of a wider range of comorbidities- including mental health. The comorbidities utilised in this study are listed in **S2**. Ethnicity was also recorded. As educational attainment has been shown to be important during recovery from critical illness, we assessed education level as: (i) university or college degree; (ii) Other professional qualification; (iii) A level/AS Level; (iv) National Vocational Qualification/ Higher National Diploma/ Higher National Certificate (v) O levels, General Certificate of Secondary Education, vocational Certificate of Secondary Education or equivalent; (vi) none of the above or prefer not to answer [20]. Full details of international qualification equivalents are shown in **S1**.

## 4 Outcomes

We examined three domains related to PICS: socio-economic, emotional and physical. Operational definitions for each domain are described below.

### 4.1 Emotional

Using a previously developed framework (**S3**), we created a measure for social isolation which was a combination of three questions in the UK Biobank [21]. We also examined self-reported loneliness, measured using a standard fixed question: ‘*Do you often feel lonely*?’ Four responses were available: ‘*Yes*’, ‘*No*’, ‘*Do not know*’ and ‘*prefer not to answer’*. Answering yes, was utilised as a positive screen.

Depression was assessed using a single question: ‘Over the past two weeks, how often have you felt down, depressed or hopeless?’. Possible answers were: ‘Not at all’, ‘several days’, ‘more than half the days’, ‘nearly every day’, ‘do not know’ and ‘prefer not to answer’. If a participant answered: several days, more than half the days, nearly every day, we categorised this as depression. Miserableness was measured by: ‘Do you ever feel ‘just miserable’ for no reason?’. Four possible responses for the answer were: ‘Yes’, ‘No’, ‘Do not know’ and ‘Prefer not to answer’. Answering yes, was utilised as a positive screen.

We examined two central concepts related to anxiety: nervousness and tension. Participants were asked: ‘*Would you call yourself a nervous person*?’ and ‘*Would you call yourself tense or highly strung*?’. For these two questions, there were four possible answers: ‘*Yes*’, ‘*No*’, ‘*Do not know’* and ‘*Prefer not to answer’*. If patients answered yes to either, they were deemed to be anxious. We also explored if patients had ever visited a General Practitioner for nerves, anxiety, tension or depression. Insomnia was measured using a standard fixed question: ‘*Do you have trouble falling asleep at night, or do you wake up in the middle of the night?*’. For this question, the response options were ‘*never/rarely’*, ‘*sometimes*’, ‘*usually*’ and ‘*prefer not to answer’*. Patients were deemed to have insomnia if they answered ‘*usually*’.

### 4.2 Social

Four social areas were examined: household income (before tax); housing tenure; employment and the use of government funded welfare support. We also assessed which participants needed a parking permit for ongoing disability.

### 4.3 Physical

Grip strength was assessed using a Hydraulic hand dynamometer of the right and left hand (Kg) in addition to Forced Vital Capacity (FVC) (Litres) [22]. Total Physical activity was computed as the sum of walking, moderate and vigorous activity, measured as metabolic equivalents (MET/min/week).

Finally, participants were asked ‘in general how would you rate your overall health?’. Answers available were: ‘*Excellent*’, ‘*good*’, ‘*fair*’, ‘*poor*’, ‘*do not know’* and ‘*prefer not to answer’*. All data fields used, alongside Biobank identifiers are provided in **S1**.

## 5 Data analysis

### 5.1 Development of cohort matching models

Due to the large volume of participants within the UK Biobank and the potential for diverse differences amongst the cohort, we chose to utilise propensity score matching using a nearest neighbour methodology to achieve a cohort with optimal common support in the analytical sample [23]. Matching was undertaken at a 1:1 ratio using age (age at hospital/critical care admission); gender; hospital diagnosis; admission type (emergency, elective, surgical (including first surgical procedure, if present) and medical); year of hospital admission; hospital length of stay (LOS); the Townsend Index; ethnicity; highest educational attainment of the participant; comorbidities; smoking status; diagnosis of alcohol abuse; presence of obesity and date between admission and UK Biobank assessment visit (from which the outcome data for this study are obtained). **S4** provides additional information on matching criteria. Differences between the two cohorts were evaluated using either Pearson Chi-Squared test or a Kruskal-Wallis test. Non-normal variables were log transformed before matching. A pre-planned analysis of early and late critical care admissions was undertaken. We choose a cut-off date of the 1st of January 2000 to differentiate the two primary cohorts, as critical care practice changed in light of emerging data around for example, neuromuscular blockade and long-term outcomes [24], [25].

### 5.2 Structural equation modelling

This study developed a Structural Equation Model (SEM). This approach is gaining momentum in the critical care field, as it concentrates on the pattern of covariation between variables and goes beyond the restrictions of univariate regression [26]. SEM is a highly flexible multivariate technique incorporating observed (measured) and unobserved (latent constructs) variables, allowing the description of often complex relationships. An advantage to this approach is the reduction in measurement error found in the use of single fixed variables [27].

The first stage of the SEM process is the creation of a hypothetical model to define latent constructs (**S5**). In this analysis, latent variables were created through clinical experience, a review of the literature, review of the data available from the UK Biobank and a recent expert consensus conference [28], [29]. We selected three latent variables to represent PICS: (i) socio-economic; (ii) physical and (iii) emotional. Cognitive data were not available for the UK Biobank for enough of the critical care group to support an analysis, thus were not included.

The SEM was fit using a diagonally weighted least squares approach with robust standard errors; this allowed the use of ordinal scores which are routinely used within the UK Biobank. Path coefficients were computed via a series of multiple regression analyses. Path diagrams (a pictorial representation of the model) were constructed with a single‐headed arrow representing the causal order between two variables, with the head pointing to the effect and the tail to the cause. A curved, double arrow was used to indicate a covariance between two variables. During SEM, standardized effects *(β* values*)* measure the direct and indirect effects of measured and latent constructs on the outcome- in this analysis this outcome was exposure to critical care. These estimated effects are standardized to critical care and correspond to effect-size estimates. To interpret: the standardised effects remove original scaling information and can be used to generate comparisons of the parameters used in the model. Larger numbers represent higher degrees of change; the scale runs from negative one to one.

We assessed SEM accuracy using three measures. First, we utilised the using Standardised Root Mean Residual (SRMR) which is an absolute measure of fit. Second, the Comparative Fit Index (CFI) measures the fit of the SEM but is not affected by model complexity. Third, the Root Mean Square Error of Approximation (RMSEA), which is related to the difference in the sample data and what would be expected if the model were assumed correct [30]. The goodness-of-fit was evaluated by the following criteria: SRMR <0.08, CFI >0.90 and RMSEA <0.06 [28]. The examination of the model included a test of the overall model fit as well as individual tests of relationships across latent domains. We undertook a sensitivity analysis on ordinal categories, including and moving individual measured variable scales, to fully understand their effect on the final SEM. We also moved measured variables between latent constructs if there was a possibility of cross over in their nature. For example, sleep could be viewed as either a physical or emotional problem, or indeed both.

All missing covariates were imputed using predictive mean matching with the Multivariate Imputation by Chained Equations (MICE) software package. each variable with missing values were regressed on all other analysed variables. Results of comparisons with a *p*-value of 0.05 or lower were considered to represent statistically significant differences. We conducted all analyses with R (Version 4.0.2). The Lavaan package (Version 0.6–6) was utilised for SEM.

The funders had no role in study design, data collection, data analysis, interpretation, writing of the report or the decision to submit for publication.

## 6 Results

### 6.1 Characteristics of cohort

From the 502,492 UK Biobank participants, 809 participants had a critical care admission with a Biobank assessment following this encounter (Fig. 1). We were able to successfully match this critical care group with 809 (100%) participants with a non-critical care hospitalisation (**S6**). Participants were admitted to critical care from 1981–2017 and underwent Biobank assessments 2006–2020. To account for the wide range of follow up time included, we created an early (pre-2000) and late (post-2000) critical care cohort and matched these with the non-critical care hospitalisation participants. The demographics of these four cohorts (critical care and matched non-critical care) are shown in Table 1. Unmatched cohort data is shown in **S7**.

**Table 1:** Characteristics of the critical care cohorts (exposure cohort) and non-critical care cohort (matched control) for the pre and post 2000 cohorts. *See S1 for full information on the meaning of the educational qualifications utilised in the study.

Demographic	Pre 2000 critical care cohort (*n* = 473)	Pre 2000 noncritical care cohort (*n* = 473)	P value	Post 2000 critical carecohort (*n* = 336)	Post 2000 noncritical care cohort (*n* = 336)	P value
Age, Years, Median (IQR)	45 (36–50)	45 (37.7–51)	0.64	61 (53–65)	60 (53–65)	0.66
Gender, Male (%)	327 (69)	312 (66)	0.3	203 (60.4)	182 (54.2)	0.1
**Ethnicity (%):**			0.72			0.86
White, British, Irish, Other	468 (99)	470 (99.4)		305 (90.8)	317 (94.3)	
Black: Caribbean	0 (0)	0 (0)		2 (0.6)	0 (0)	
Black: African	0 (0)	0 (0)		1 (0.3)	1 (0.3)	
Indian	0 (0)	0 (0)		13 (3.8)	8 (2.4)	
Pakistani	5 (1)	3 (0.6)		1 (0.3)	0 (0)	
Other: South Asian	0 (0)	0 (0)		4 (1.2)	3 (0.9)	
Mixed	0 (0)	0 (0)		1 (0.3)	1 (0.3)	
Chinese	0 (0)	0 (0)		1 (0.3)	1 (0.3)	
Other	0 (0)	0 (0)		8 (2.4)	5 (1.5)	
Comorbidities (2 or more) (%)	91 (19.2)	65 (13.7)	0.02	188 (56)	168 (50)	0.12
*Highest Education Attainment (%):			0.5			0.84
College/University Degree	124 (26.2)	150 (31.7)		92 (27.4)	107 (31.8)	
Other professional qualification	55 (11.7)	54 (11.4)		52 (15.5)	48 (41.3)	
A Levels/ AS Levels	28 (5.9)	30 (6.3)		23 (6.8)	21 (6.3)	
NVQ/HND/HNC	35 (7.4)	38 (8.1)		38 (11.3)	29 (8.6)	
O Levels/GCSEs/CSEs	43 (9.7)	49 (10.4)		61 (18.2)	60 (17.9)	
None of the above/Prefer not to answer	185 (39.1)	152 (32.1)		70 (20.8)	71 (21.1)	
Townsend Index, Median (IQR)	−0.4 (−3.21–3.26)	−0.73 (−3.18–2.82)	0.54	−1.28 (−3.37–2.26)	−1.61 (−3.44–1.94)	0.29
**Smoking Status (%):**			0.38			0.99
Prefer not to answer	2 (0.5)	3 (0.6)		3 (0.9)	3 (0.9)	
Current	99 (20.9)	91 (19.2)		37 (11)	37 (11)	
Previous	194 (41)	176 (37.3)		147 (43.8)	151 (44.9)	
Never	178 (37.6)	203 (42.9)		149 (44.3)	145 (43.2)	
Days between critical care/ hospitalisation and assessment, Median (IQR)	5870 (4783–7199)	5920 (4669–7553)	0.74	628 (336–1093)	640 (305–1100)	0.45
Admission Type, Surgical (%)	153 (32.3)	159 (33.6)	0.68	290 (86.3)	279 (83.3)	0.24
Admission Type, Emergency (%)	432 (91.3)	432 (92)	0.72	184 (54.8)	196 (58.3)	0.35
Hospital Length of Stay, days, Median (IQR)	6 (2–10)	4 (2–8)	<0.01	10 (5–20)	8 (4–18)	0.01
Obesity (Previous/Current Diagnosis) (%)	3 (0.6)	2 (0.4)	0.65	25 (7.4)	20 (6)	0.44
Alcohol Abuse (Previous/Current Diagnosis) (%)	16 (3.4)	12 (2.5)	0.44	31 (9.2)	22 (6.5)	0.2

473 of the critical care cohort were admitted pre-2000. In the pre-2000 critical care cohort, the median age was 45 years (42–49 IQR), 372 (69%) were men and 91 (19.2%) had two or more comorbidities. In the pre-2000 propensity matched cohort, the median age was 45 years (37.7–51 IQR), 312(66%) were men and 65 (13.7%) had two or more comorbidities (Table 1). Across the both the pre-2000 and the post-2000 cohorts, 9.4% of participants had two or more critical care admissions, before their UK Biobank assessment visit.

Compared with the matched participants who had been hospitalised without critical care, more participants in the pre-2000 critical care group were classified as socially isolated (10 (2.1%) vs 1 (0.2%), *p* *=* 0.01) and more had symptoms of anxiety such as tension (123 (20%) vs 88 (18.6%), *p* = 0.02). A higher number of the critical care cohort were in receipt of government funded welfare support (110 (23.3%) vs 63 (13.3%), *p* < 0.01) (Table 2).

**Table 2:** Outcomes of the critical care cohorts and matched control for the pre and post 2000 cohorts.

Outcome	Pre-2000 critical care cohort (*n* = 473)	Pre-2000 noncritical care cohort (*n* = 473)	P value	Post-2000 critical carecohort (*n* = 336)	Post-2000 noncritical care cohort (*n* = 336)	P value
Insomnia, n (%)	170 (35.9)	152 (32.1)	0.42	116(34.5)	87 (28.9)	0.26
Social Isolation n, (%)	10 (2.1)	1 (0.2)	**0.01**	8 (2.4)	3 (0.9)	0.13
Depression, n (%)	146 (31)	141 (29.8)	0.49	108 (32.1)	80 (23.8)	**<0.01**
Loneliness, n (%)	102 (21.6)	103 (21.8)	0.82	94 (28)	66 (19.6)	**0.02**
Miserableness, n (%)	198 (41.9)	174 (36.8)	0.46	153 (45.5)	132 (39.3)	0.32
Nervousness, n (%)	144 (30.4)	122 (25.8)	0.17	93 (27.7)	65 (19.3)	**0.02**
Tense/highly strung (%)	123 (26)	88 (18.6)	**0.02**	78 (23.2)	67 (19.9)	0.59
Primary care use for mental health issues, n (%)	178 (37.6)	174 (36.8)	0.35	147 (43.8)	109 (32.4)	**0.01**
**Household income (%)**			0.09			0.37
Less than £18,000	169 (35.7)	152 (32.1)		100 (29.8)	95 (28.3)	
£18,000-£30,999	81 (17.1)	106 (22.4)		79 (23.5)	63 (18.8)	
£31,000-£51,999	98 (20.7)	72 (15.2)		56 (16.7)	73 (21.7)	
£52,000-£100,000	59 (12.5)	70 (14.8)		35 (10.4)	40 (11.9)	
Greater than £100,000	23 (4.9)	27 (5.7)		21 (6.3)	16 (4.7)	
Prefer not to answer	43 (9.1)	46 (9.7)		45 (13.3)	49 (16.6)	
**Housing Tenure**			0.26			0.12
Own outright	252 (53.3)	248 (52.4)		186 (55.4)	198 (58.9)	
Own with mortgage	115 (24.3)	139 (29.4)		79 (23.5)	92 (27.4)	
Rent: Local authority	80 (16.9)	71 (15)		43 (12.8)	29 (8.6)	
Rent: Private Landlord	14 (3.0)	7 (1.5)		15 (4.5)	14 (4.2)	
Part Rent/Part Mortgage	2 (0.4)	1 (0.2)		2 (0.6)	0 (0)	
Rent free accommodation	6 (1.3)	5 (1.1)		3 (0.8)	0 (0)	
None of the above/prefer not to answer	4 (0.8)	2 (0.4)		8 (2.4)	3 (0.9)	
**Employment Status**			0.13			0.12
Not defined	2 (0.4)	3 (0.6)		5 (1.5)	2 (0.6)	
Unable to work	74 (15.6)	50 (10.6)		56 (16.7)	38 (11.3)	
Vocational	3 (0.6)	4 (0.8)		1 (0.3)	2 (0.6)	
Purposeful	394 (83.3)	416 (87.9)		274 (81.5)	294 (87.5)	
**Government funded support**			**<0.01**			0.02
Allowances (Welfare Benefits)	110 (23.3)	63 (13.3)		65 (19.3)	43 (12.8)	
No allowances	347 (73.4)	398 (84.1)		257 (76.5)	285 (84.8)	
Blue Badge (Parking assistance)	16 (3.4)	12 (2.5)		14 (4.2)	8 (2.4)	
Right Hand- Grip Strength, Kg, Median (IQR)	34 (26–42)	36 (27–43)	0.35	29 (22–36)	30 (22–38)	0.19
Left Hand-Grip Strength, Kg, Median (IQR)	32 (23–42)	32 (24–40)	0.67	26 (20–34)	28 (20–36)	0.18
Forced Vital Capacity, Litres, Median (IQR)	3.76 (2.91–5.29)	3.68 (2.94–4.69)	0.29	3.27 (2.68–3.89)	3.29 (2.71–4.01)	0.28
MET (minutes per week), Median (IQR)	65 (15–140)	80 (20–160)	0.11	100 (50–256)	110 (50–240)	0.9
**Overall Health Rating**			**0.03**			**<0.01**
Prefer not to answer	1 (0.2)	0 (0)		1 (0.3)	2 (0.6)	
Poor	84 (17.8)	52 (11.2)		75 (22.3)	26 (7.8)	
Fair	155 (32.8)	151 (31.9)		110 (32.7)	117 (34.8)	
Good	197 (41.6)	227 (48)		128 (38.1)	160 (47.6)	
Excellent	36 (7.6)	42 (8.9)		22 (6.6)	31 (9.2)	

336 patients in the UK Biobank were admitted to critical care after 1st of January 2000. In the post-2000 critical care cohort, the median age was 61 years (53–65 IQR), 303 (60.4%) were men and 188 (56%) had two or more comorbidities. In the propensity score matched cohort, the median age was 60 years (53–65 IQR), 182 (54.2%) were men and 168 (50%) had two or more comorbidities (Table 1). Compared with the matched participants, hospitalised without critical care, more participants in the post-2000 critical care group were classified as depressed (108 (32.1%) vs 80 (23.8%), *p* *<* *0.01*), and more patients had sought help from primary care providers for mental health problems (147 (43.8%) vs 109 (32.4%), *p* = 0.02). A higher number were in receipt of government funded welfare support (65 (19.3%) vs 43 (12.8%), *p* = 0.02) and the critical care group also had poorer self-reported health (*p* = 0.01) (Table 2).

## 7 Interrelationships among outcomes

### 7.1 Pre-2000 cohort

**Fig. 2:**
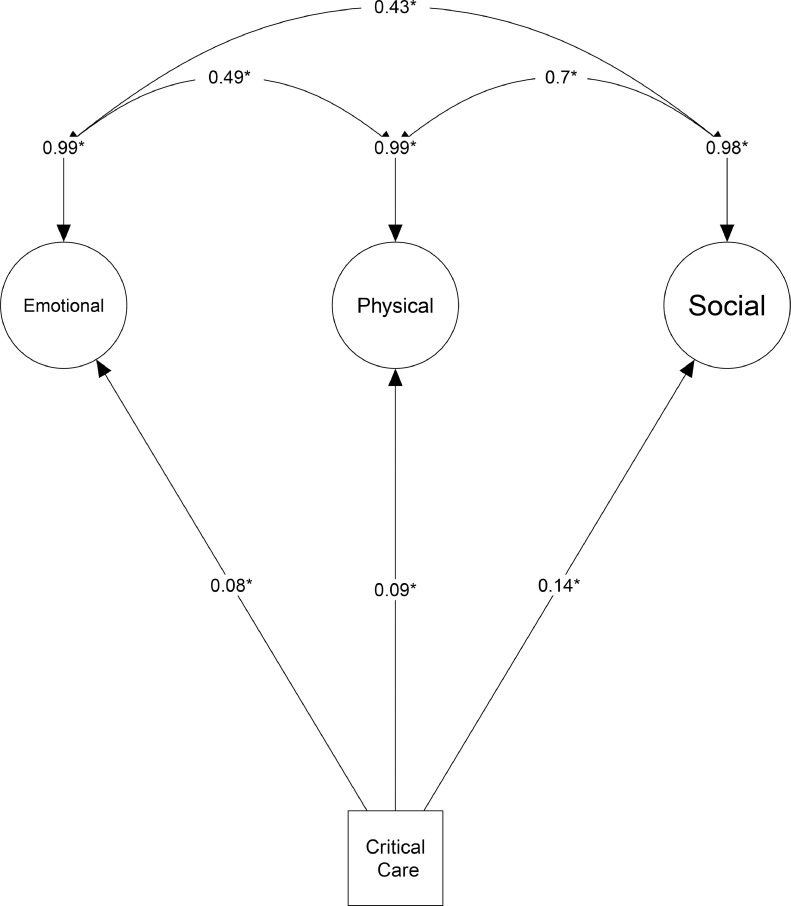
Pre-2000 Structural Equation Model, with the inclusion of physical domain.

The analysis and testing of the different hypothesized associations with SEM resulted in the model presented in Fig. 2. All hypothesized associations were confirmed outwith self-perceived health rating and insomnia **(S5)**, which moved across latent domains in the final model.

Model fit parameters for this SEM were: SRMR: 0.064, CFI: 0.912, RMSEA: 0.065 (95% CI: 0.06–0.071). There was a significant correlation between exposure to critical care (compared to other hospitalizations) and poorer outcomes in the three latent domains created: emotional (*β=*0.08 (95% CI: 0.00–0.15), *p* = 0.049), physical (*β=*0.09 (95% CI:0.01–0.17), *p* = 0.047) and socio-economic (*β* =0.14, (95% CI:0.06–0.23) *p* < 0.002). There was a statistically significant relationship found between all three latent domains: between emotional and physical (*β*=0.49 (95% CI: 0.40–0.58), *p* < 0.001), emotional and socio-economic (*β*=0.43 (95% CI:0.33–0.54), *p* < 0.001) and physical and socio-economic (*β*=0.70 (95% CI: 0.61–0.80), *p* < 0.001) (Fig. 2).

### 7.2 Post-2000 cohort

**Fig. 3:**
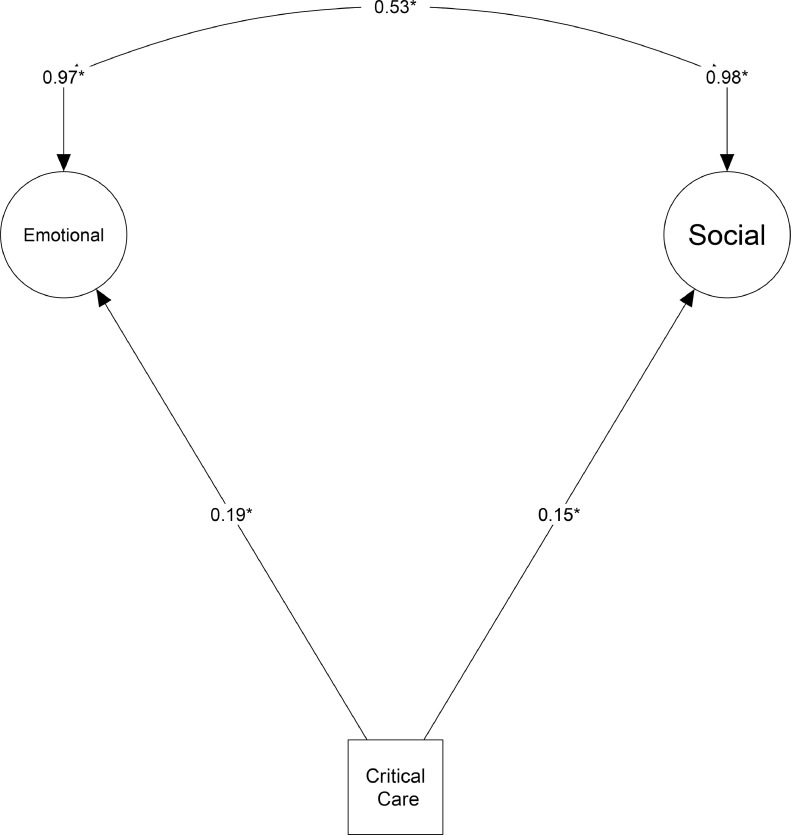
Post-2000 Structural Equation Model with the removal of the physical domain.

A significant correlation was found between exposure to critical care (compared to other hospitalisations) and poorer outcomes in the two domains: emotional (*β*=0.16 (95% CI: 0.03–0.30, *p* = 0.001) and socio-economic (*β* =0.15 (95% CI: 0.04–0.26), *p* < 0.009) (Fig. 3). There was no difference in physical outcomes between the critical care cohort and non-critical care hospitalisation cohort (*β* =0.09 (95% CI: −0.80–0.97), *p* = 0.25) in the more recent timeframe. Model fit parameters also dropped: SRMR: 0.071, CFI: 0.869, RMSEA: 0.069 (95% CI: 0.062–0.076) in the post-2000 SEM, demonstrating that the model no longer represented the integrated model hypothesised. There was a difference between the time periods (early vs. late) for the physical latent (*p* *=* *0.009*), but not the emotional (*p* *=* *0.61*) or socio-economic (*p* *=* *0.24*).

The post-2000 PICS model was analysed without a physical domain. This resulted in the movement of self-perceived health rating back into the emotional domain (as per the original hypothesised model). The fit for this newly created SEM was: SRMR: 0.044, CFI: 0.982, RMSEA: 0.026 (95% CI: 0.013–0.036), representing an excellent fit for the data. There was a significant correlation between exposure to critical care and poorer outcomes in emotional (*β* =0.19 (95% CI:0.10–0.27), *p* < 0.001) and socio-economic (*β=* 0.15 (95%: 0.05–0.25), *p* = 0.007) domains (Table 3). There was a significant relationship between the two domains: (*β*=0.53 (95% CI: 0.40–0.66), *p* < 0.001) (Fig. 3).

**Table 3:** Regression weights between structural parameters for the pre-2000 cohort, alongside the newly created post-2000 SEM. Standardised effects remove original scaling information and can be used for comparisons. Larger numbers represent higher degrees of change; the scale runs from negative one to one.

	Unstandardised effects	Standard Error	Critical Ratio	P value	Standardised effect (*β)* (95% CI)
**Pre-2000 SEM**					
**Emotional**					
Nervous feelings	1				0.47 (0.39–0.54)
Primary Care for mental health issues	1.23	0.12	10.09	<0.001	0.53 (0.47–0.59)
Depression	2.27	0.25	9.07	<0.001	0.65 (0.58–0.72)
Tense/Highly Strung	1.09	0.09	12.11	<0.001	0.53 (0.46–0.60)
Loneliness	1.02	0.12	8.66	<0.001	0.51 (0.44–0.58)
Miserableness	1.46	0.15	9.5	<0.001	0.62 (0.55–0.68)
Social Isolation	0.001	0.01	0.13	0.897	0.00 (−0.04–0.05)
Insomnia	0.62	0.18	3.56	0.211	0.18 (0.08–0.27
Critical Care	0.03	0.02	1.97	0.049	0.08 (0.00–0.15)
**Physical**					
Insomnia	1				0.27 (0.18–0.37)
Left Hand- Grip Strength	−2.58	0.51	−5.1	<0.001	−0.52 (−0.58- −0.45)
Right Hand- Grip Strength	−2.50	0.5	−5	<0.001	−0.50 (−0.57- −0.42)
FVC	−2.71	0.53	−5.13	0.114	−0.54 (−0.62- −0.46)
Overall health rating	−2.56	0.5	−5.17	<0.001	−0.61 (−0.68- −0.54)
Critical Care	0.04	0.02	1.98	0.047	0.09 (0.01–0.17)
**Socio-economic**					
Government funded support	1				0.57 (0.50–0.64)
Employment Status	−0.63	0.08	−7.77	<0.001	−0.40 (−0.48- −0.32)
Household Income	−1.55	0.17	−9.28	<0.001	−0.59 (−0.65- −0.62)
Housing Tenure	0.85	0.12	7.4	<0.001	0.38 (0.30–0.46)
Critical Care	0.13	0.04	3.09	0.002	0.14 (0.06–0.23)
**Post-2000 SEM (without physical latent)**					
**Emotional**					
Nervous Feelings	1				0.46 (0.37–0.54)
Primary care for Mental Health Issues	1.20	0.14	8.85	<0.001	0.48 (0.41–0.55)
Depression	2.84	0.36	7.91	<0.001	0.62 (0.54–0.70)
Tense/highly strung	1.11	0.1	10.73	<0.001	0.52 (0.43–0.60)
Loneliness	1.27	0.14	8.77	<0.001	0.58 (0.50–0.65)
Miserableness	1.41	0.16	8.81	<0.001	0.55 (0.49–0.62)
Social isolation	0.13	0.05	2.59	0.01	0.19 (0.08–0.31)
Insomnia	1.30	0.2	6.59	<0.001	0.35 (0.27–0.43)
Self-perceived health	−1.99	0.26	−7.8	<0.001	−0.46 (−0.54- −0.39)
Critical Care	0.07	0.02	3.91	<0.001	0.19 (0.10–0.27)
**Socio-economic**					
Government funded support	1				0.45 (0.31–0.58)
Employment Status	−1.39	0.25	−5.56	<0.001	−0.62 (−0.74- −0.49)
Household Income	−1.29	0.29	−4.38	<0.001	−0.34 (−0.43- −0.24)
Housing Tenure	1.25	0.28	4.41	<0.001	0.41 (0.30–0.53)
Critical Care	0.10	0.04	2.72	0.007	0.15 (0.05—0.25)

Full SEM models for the pre and post-2000 analyses, including all observed and unobserved variables, are shown in **S8.**

## 8 Discussion

This cohort study has found a significant difference in emotional and social outcomes between critical care and non-critical care hospitalised participants in the UK Biobank, after matching for individual demographics, comorbidities and nature of hospitalisation.

Despite significant differences in social and emotional outcomes, physical outcomes such as FVC and grip strength were not significantly different between the critical care and non-critical care hospitalised group in the post-2000 timeframe. The post-2000 cohort were older and more comorbid than the pre-2000 cohort, a trend which is reflected in critical care admissions internationally [31], [32], [33]. Year on year critical care use is being extended to patients with more complex comorbidities and those who are older and frailer, thus outcome measures such as FVC and grip strength may lack the sensitivity may lack the sensitivity to detect differences [34], [35]. We hypothesise that physical problems or disability which can occur as a result of critical care have not disappeared. Instead, this finding is consistent with recent work which demonstrates that the physical outcomes measurements currently utilised are unlikely to be appropriate [36], [37]. Changes in practice seen in post-2000 critical care, such as early mobilisation programmes and the implementation of innovations such as the ABCDEF bundle, may also account for these differences [38]. Future work is urgently required into the measurement and conceptualisation of physical problems following critical illness.

Previous research has attempted to address the needs of critical care survivors through rehabilitation programmes [7,10,39]. These programmes have predominately focused on emotional and physical problems and have seen limited success. This analysis, consistent with previous work, has demonstrated that socio-economic problems are common and are intrinsically linked with emotional health [40], [41]. Moreover, the domains of PICS do not exist in isolation and clinicians must pay careful attention to all aspects of wellbeing if gains in health are to be made. Future research should focus on the delivery of support which facilitates social re-integration and economic wellbeing- especially considering the global economic problems emerging from the COVID-19 pandemic. Such interventions have been tested previously, have proven feasible have been deemed acceptable by patients [42], [43].

The UK Biobank has provided an opportunity to examine outcomes following critical illness in a large national prospective cohort. The response rate to the UK Biobank was only 5.5%; although it is representative of the UK general population with respect to age, ethnicity and deprivation [43]. However, it may not be representative across other characteristics and caution must be taken with the interpretation of these results. The UK Biobank data set does not contain core outcome measures which are recommended in critical care research [36]. Single fixed questions were used as surrogates for problems such as anxiety and depression, as such we may have mis-classified problems in some participants. Although cognitive data are available within the UK Biobank, we were unable to utilise them as the ‘case’ cohort did not have enough information available. Despite this, the SEM approach has demonstrated that there is a significant difference in some measured domains of PICS between non-critical care and critical care admissions. We classified patients as having a critical care exposure based on Consultant care, this may have led to the misclassification of a small number of participants in this dataset. Although we have information on admission type, we have limited data about the severity of illness of participants, as these data were not available for this study. This dearth of data does not allow us to contextualise admission decisions or the critical care journey. Finally, although we matched on a range of characteristics for the hospital and critical care cohorts, the critical care cohort had more mental health problems and had a greater dependency on government assistance at baseline. The constraints of the UK Biobank data do not allow for more detailed analysis of baseline characteristics; therefore, critical care exposure may not be the only explanation for the results presented. This may have directly impacted the outcomes reported and is a significant limitation.

## 9 Conclusion

Using a large prospectively collected cohort, we have shown that survivors of critical illness have different psycho-social outcomes to those patients hospitalised without critical care. Our findings suggest that critical care patients may benefit from enhanced support across health and social care boundaries.

## Declarations

### Funding

JM is funded by a THIS.Institute (University of Cambridge) Research Fellowship (PD-2019–02–16).

AHL is part of the Social and Public Health Sciences Unit, funded by the Medical Research Council (MC_UU_12,017/13) and the Scottish Government Chief Scientist Office (SPHSU13).

## Ethics approval

The UK Biobank study was approved by the North West Multicentre Ethics Research Committee; participants provided written informed consent for data collection and analysis. This study is part of UK Biobank project 57617 (NHS National Research Ethics Service Ref: 11/NW/0382).

## Consent to participate

N/A

## Consent to publication

N/A

## Availability of material

The data that support the findings of this study are available from the UK Biobank (http://ukbiobank.ac.uk), but restrictions apply to their availability.

## Conflicts of Interest

The authors have no conflicts of interest to declare. This research was designed, conducted, analysed and interpreted by the authors entirely independently of the funding sources.

## Appendix Supplementary materials

Image, application 1

Image, application 2

Image, application 3

Image, application 4

Image, application 5

## References

[1] McPeake, J.M. Boehm, L.M. Hibbert, E.B. (2020) Key Components of ICU Recovery programs: what did patients report provided benefit?*Critic Care Explorat*; 2(4): e0088. 10.1097/CCE.0000000000000088 PMC7188426 32426730

[2] Prescott, H.C. Angus, D.C. (2018) Enhancing recovery from sepsis: a review. *J Am Med Assoc*;319(1):62–75. 10.1001/jama.2017.17687 PMC5839473 29297082

[3] Iwashyna, T.J. Ely, E.W. Smith, D.M. et al. (2010) Long Term cognitive impairment and functional disability among survivors of severe sepsis. *J Am Med Assoc*; 304(16):1787–1794. 10.1001/jama.2010.1553 PMC3345288 20978258

[4] Wade, D.M. Howell, D.C. Weinman, J.A. et al. (2012) Investigating risk factors for psychological morbidity three months after intensive care: a prospective study. *Crit Care;*16: R192. 10.1186/cc11677 PMC3682294 23068129

[5] Needham, D.M. Davidson, J. Cohen, J. et al. (2012) Improving long-term outcomes after discharge from intensive care unit. *Crit Care Med*;40(2):502–509. 10.1097/CCM.0b013e318232da75 21946660

[6] McPeake, J.M. Mikkelsen, M.E. (2018) The evolution of post intensive care syndrome. *Crit Care Med*;46(9):1551–1552. 10.1097/CCM.0000000000003232 30113373

[7] Walsh, TS.Salisbury, L.G. Merriweather, J.L. Et al (2015) Increased hospital-based physical rehabilitation and information provision after intensive care unit discharge: the RECOVER randomized clinical trial. *JAMA Internal Med.*; 175(6):901–910. 10.1001/jamainternmed.2015.0822 25867659

[8] Sevin C.M., Bloom S.L., Jackson J.C., et al. (2018) Comprehensive care of ICU survivors: development and implementation of an ICU recovery center. *J Critic Care*; 46:141–148 10.1016/j.jcrc.2018.02.011 PMC6020044 29929705

[9] McPeake J., Iwashyna T.J., Devine H., et al. (2017) Peer support to improve recovery following critical care discharge: a case-based discussion. *Thorax*; 72(9): 856–8. 10.1136/thoraxjnl-2016-209661 28213591

[10] Schofield-Robinson O.J., Lewis S.R., Smith A.F., et al. (2018) Follow-up service for improving long-term outcomes in intensive care (ICU) survivors. *Cochrane Database Systematic Rev.*; 11:1465–1858. 10.1002/14651858.CD012701.pub2 PMC6517170 30388297

[11] Feemster, LC.Cooke, C.R. Rubenfeld, G.D. Et al (2015). The influence of hospitalisation or intensive care unit admission on declines in health-related quality of life. *Annal Am Thoracic Soc.*;12(1):35–45. 10.1513/AnnalsATS.201404-172OC PMC4342801 25493656

[12] Krumholz, H.M. (2013) Post Hospital Syndrome- an acquired, transient condition of generalised risk. *N Engl J Med*; 368:100–102. 10.1056/NEJMp1212324 PMC3688067 23301730

[13] Ferguson, L.D. Brown, R. Celis-Morales, C. et al. (2019) Association of central adiposity with psoriasis, psoriatic arthritis and rheumatoid arthritis: a cross sectional study of the UK Biobank. *Rheumatology*; 58:2137–2142. 10.1093/rheumatology/kez192 PMC6880847 31131407

[14] Sudlow C., Gallacher J., Allen N. et al. (2015) UK Biobank: an open access resource for identifying the causes of a wide range of complex diseases of middle and old age. *PLoS Med.*;12: e1001779. 10.1371/journal.pmed.1001779 PMC4380465 25826379

[15] UK Data Service Census Data (2017) 2011 UK townsend deprivation score. Accessed 24th of October 2020: https://www.statistics.digitalresources.jisc.ac.uk/dataset/2011-uk-townsend-deprivation-scores.

[16] Elixhauser, A. Steiner, C. Harris, D.R. Coffey, R.M. (1998) Comorbidity measures for use with administrative data. *Med Care*;36(1):8–27. 10.1097/00005650-199801000-00004 9431328

[17] Sundararajan, V. Henderson, T. Perry, C. et al. (2004) New ICD-10 version of the Charlson comorbidity index predicted in hospital mortality. *J Clin Epidemiol*;57(12):1288–1294. 10.1016/j.jclinepi.2004.03.012 15617955

[18] Ho, K.M. Finn, J. Knuiman, M. et al. (2007) Combining multiple comorbidities with Acute Physiology Score to predict hospital mortality of critically ill patients: a linked data cohort study. *Anaesthesia*; 62:1095–1100. 10.1111/j.1365-2044.2007.05231.x 17924888

[19] Nielsen, A.B. Hans-Christian, T.M. Belling, K. et al. (2019) Survival prediction in intensive-care units based on aggregation of long-term disease history and acute physiology: a retrospective study of the Danish National Patient Registry ad electronic patient records. *Lancet Digital Health*; 1(2): E78–89. 10.1016/S2589-7500(19)30024-X 33323232

[20] Marra, A., Pandharipande, P.P. Girad, T.D. et al. (2018) Co-occurrence of post-intensive care syndrome problems among 406 survivors of critical illness. *Crit Care Med*;46(9):1393–1401. 10.1097/CCM.0000000000003218 PMC6095801 29787415

[21] Morneau-Vaillancourt, G. Coleman, J.R.I. Purves, K.L. et al. (2019) The genetic and environmental hierarchical structure of anxiety and depression in the UK Biobank. *Depression Anxiety*; 37:512–520. 10.1002/da.22991 PMC7318128 31951317

[22] Celis-Morales, C.A. Lyall, D.M. Anderson, J. et al. (2017) The association between physical activity and risk of mortality is modulated by grip strength and cardiorespiratory fitness: evidence from 498135 UK-Biobank participants. *Eur Heart J;*38:116–122. 10.1093/eurheartj/ehw249 PMC5837781 28158566

[23] Desai, R.J. Franklin, J.M. (2019) Alternative approaches for confounding adjustment in observational studies using weighting based on the propensity score: a primer for practitioners. *Brit Med J*; 367:15657. 10.1136/bmj.l5657 31645336

[24] The Acute Respiratory Distress Syndrome Network (2000) Ventilation with lower tidal volumes as compared with traditional tidal volumes for acute lung injury and the acute respiratory distress syndrome. *N Engl J Med*; 342:1301–1308. 10.1056/NEJM200005043421801 10793162

[25] Herridge, M.S. Cheung, A.M. Tansey, C.M. et al. (2003) One-year outcomes in survivors of the acute respiratory distress syndrome. *New Engl J Med*; 348:683–693. 10.1056/NEJMoa022450 12594312

[26] Hurley, J.C. (2020) Structural equation modelling the ‘control of gut overgrowth’ in the prevention of ICU acquired gram-negative infection. *Crit Care*;24;189. 10.1186/s13054-020-02906-6 PMC7199305 32366267

[27] Dion, P.A. (2008) Interpreting structural equation modelling results: a reply to martin and Cullen. *J Bus Ethics;*83 (3):356–368.

[28] Mikkelsen M.E., Still M., Anderson B.J., et al. (2020). Society of critical care Medicine's international consensus conference on prediction and identification of long-term impairments after critical illness. *Crit Care Med*; 2020: In press.

[29] Haines, K.J. Hibbert, E. McPeake, J.M. (2020) Prediction models for physical, cognitive and mental health impairments after critical illness: a systematic review and critical appraisal. *Crit Care Med*;48(12):1871–1880. 10.1097/CCM.0000000000004659 PMC7673641 33060502

[30] Kline, R.B. (2015) *Principles and practice of structural equation modelling*. New York. Guilford Press.

[31] Barnato, AE.Albert, S.M. Angus, D.C. Et al (2011) Disability among elderly survivors of mechanical ventilation. *Am J Respirator Crit Care Med*; 183:1037–1042. 10.1164/rccm.201002-0301OC PMC3159078 21057004

[32] Simpson, A. Puxty, K. McLoone, P. et al. (2020) Comorbidity and survival after admission to the intensive care unit: a population-based study of 41230 patients. *J Intensive Care Soc*(published early online): https://doi.org/10.1177%2F1751143720914229. 10.1177/1751143720914229 PMC8120566 34025754

[33] Guidet, B. Vallet, H. Boddaert, J. et al. (2018) Caring for the critically ill patients over 80: a narrative review. *Annal. Intensive Care*;8;114. 10.1186/s13613-018-0458-7 PMC6261095 30478708

[34] Bagshaw, SM.Majumdar, S.R. Rolfson, D.B. Et al (2016) A prospective multicentre cohort study of frailty in younger critically ill patients; *Critic Care*; 20:175. 10.1186/s13054-016-1338-x PMC4893838 27263535

[35] Muscedere, J. Warers, B. Varambally, A. et al. (2017) The impact of frailty on intensive care unit outcomes: a systematic review and meta-analysis. *Intensive Care Med*; 43:1105–1122. 10.1007/s00134-017-4867-0 PMC5501903 28676896

[36] Needham, D.M. Sepulveda, K.A. Dinglas, V.D. et al. (2017) Core Outcome measures for clinical research in Acute Respiratory Failure survivors. An international modified Delphi consensus study. *Am J Respirator Crit Care Med*;196(9):1122–1130. 10.1164/rccm.201702-0372OC PMC5694837 28537429

[37] Chan, K.S. Friedman, L.A. Diglas, V.D. et al. (2016) Are physical outcome measures related to patient-centred outcomes in ARDS survivors?*Thorax*; 72:884–892. 10.1136/thoraxjnl-2016-209400 PMC6888961 28108621

[38] Ely, W.E. (2017) The ABCEDF Bundle: science and philosophy of how ICU liberation serves patients and families. *Crit Care Med*;45(2):321–330. 10.1097/CCM.0000000000002175 PMC5830123 28098628

[39] Cuthbertson, B. Rattray, J. Campbell, M.K. et al. (2009) The PRaCTICaL study of nurse led, intensive care follow-up programmes for improving long term outcomes from critical illness: a pragmatic randomised controlled trial. *Brit Med J*;339: b3723. 10.1136/bmj.b3723 PMC2763078 19837741

[40] Hauschildt, K.E. Seigworth, C. Kamphuis, L.A. et al. (2020). Financial toxicity after acute respiratory distress syndrome. A national qualitative cohort study. *Crit Care Med*; doi10.1097/CCM. 10.1097/CCM.0000000000004378 PMC7387748 32697479

[41] McPeake, J. Mikkelsen, M.E. Quasim, T. et al. (2019) Return to Employment after Critical Illness and Its Association with psychosocial outcomes: a systematic review and meta-analysis. *Annal Am Thoracic Soc*;16(10): 1304–1311. 10.1513/AnnalsATS.201903-248OC 31184500

[42] McPeake, J.M. Henderson, P. Darroch, G. et al. (2019) Social and economic problems of ICU survivors identified by a structured welfare consultation. *Crit Care*; 23:153. 10.1186/s13054-019-2442-5 PMC6498562 31046813

[43] Fry, A. Littlejohns, T.J. Sudlow, C. et al. (2017) Comparison of sociodemographic and health-related characteristics of UK Biobank participants with those of the general population. *Am J Epidemiol*; 186: 1026–1034. 10.1093/aje/kwx246 PMC5860371 28641372

