# Supplementary figures and images for: Long term outcomes following critical care hospital admission: A prospective cohort study of UK biobank participants
Source: Lancet Reg Health Eur. 2021 Jun 15;6:100121. doi: 10.1016/j.lanepe.2021.100121 (PMC8278491; doi:10.1016/j.lanepe.2021.100121)

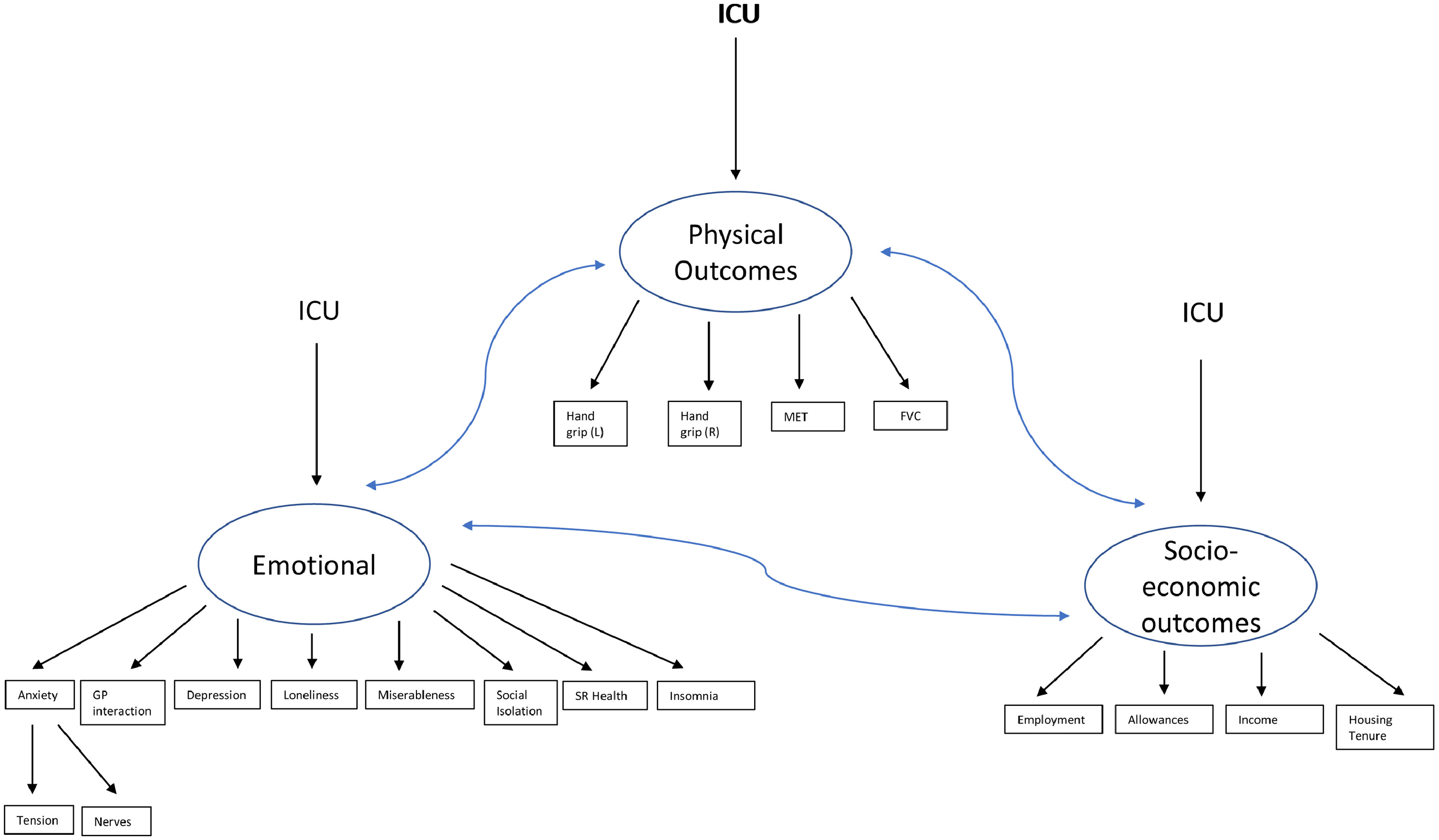

Supplement: Supplementary file 1 [file mmc1.jpg]

## Pre 2000

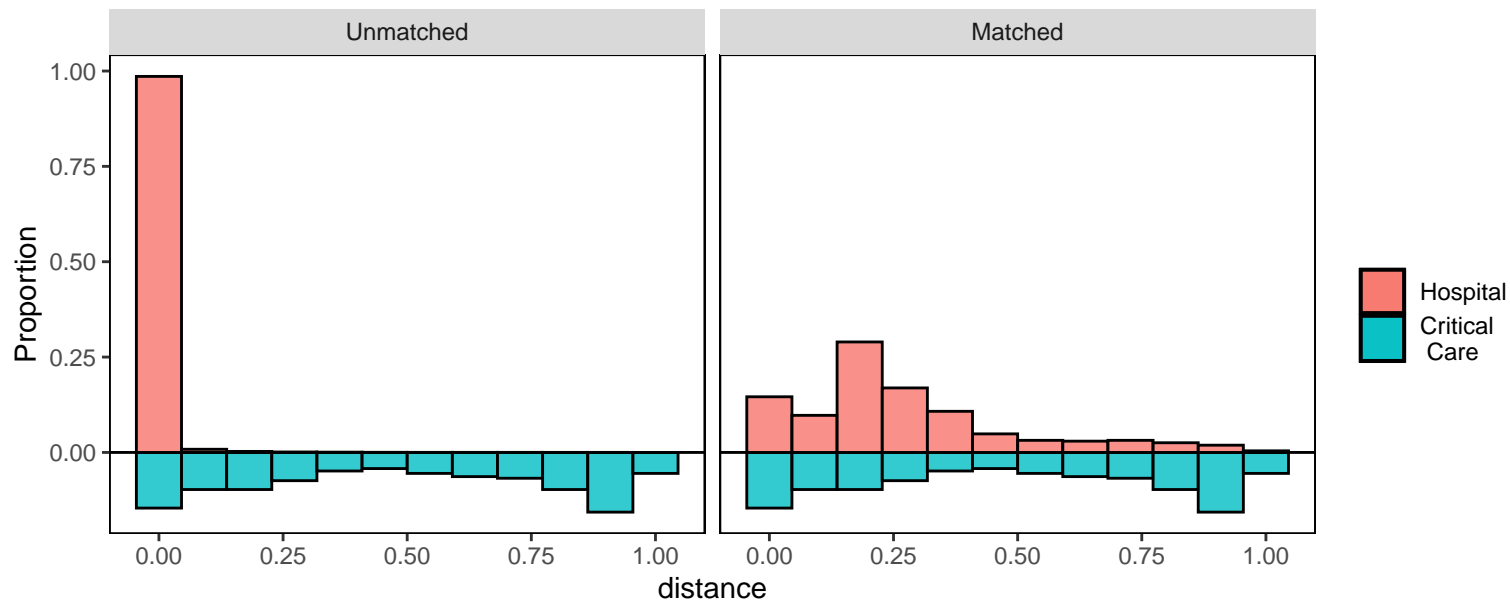

## Post 2000

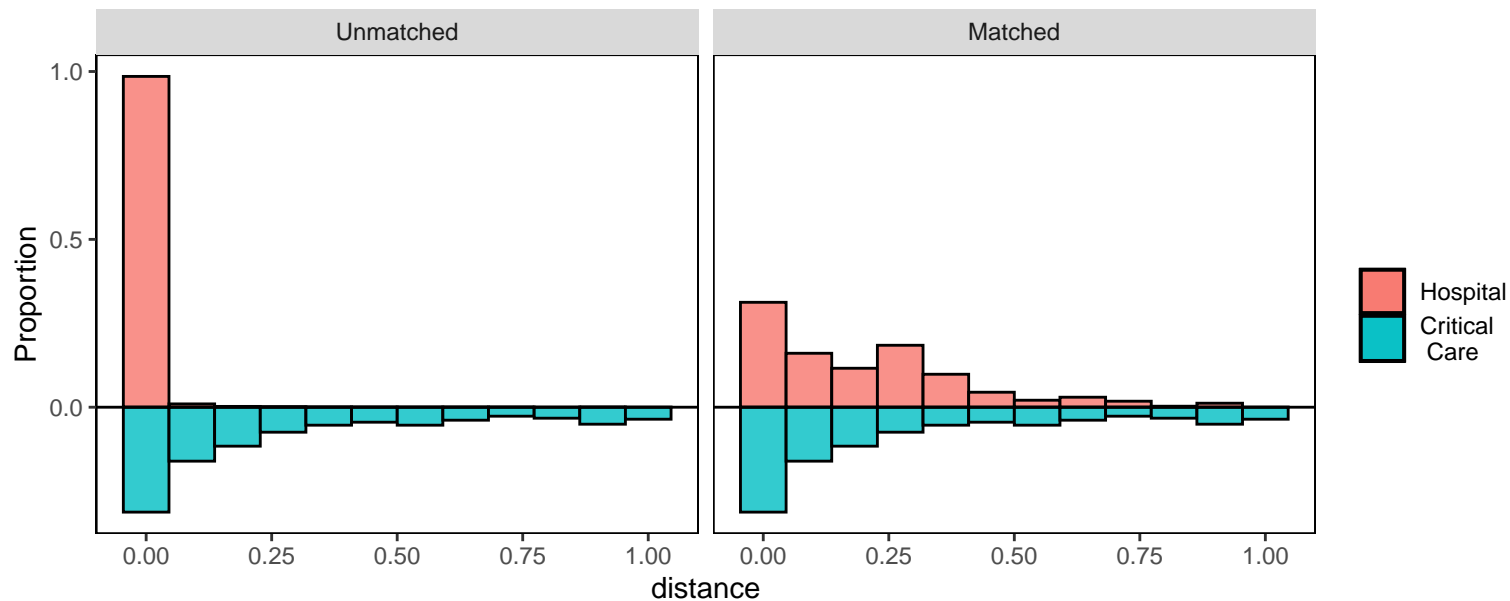

Supplement: Supplementary file 2 [file mmc2.pdf]

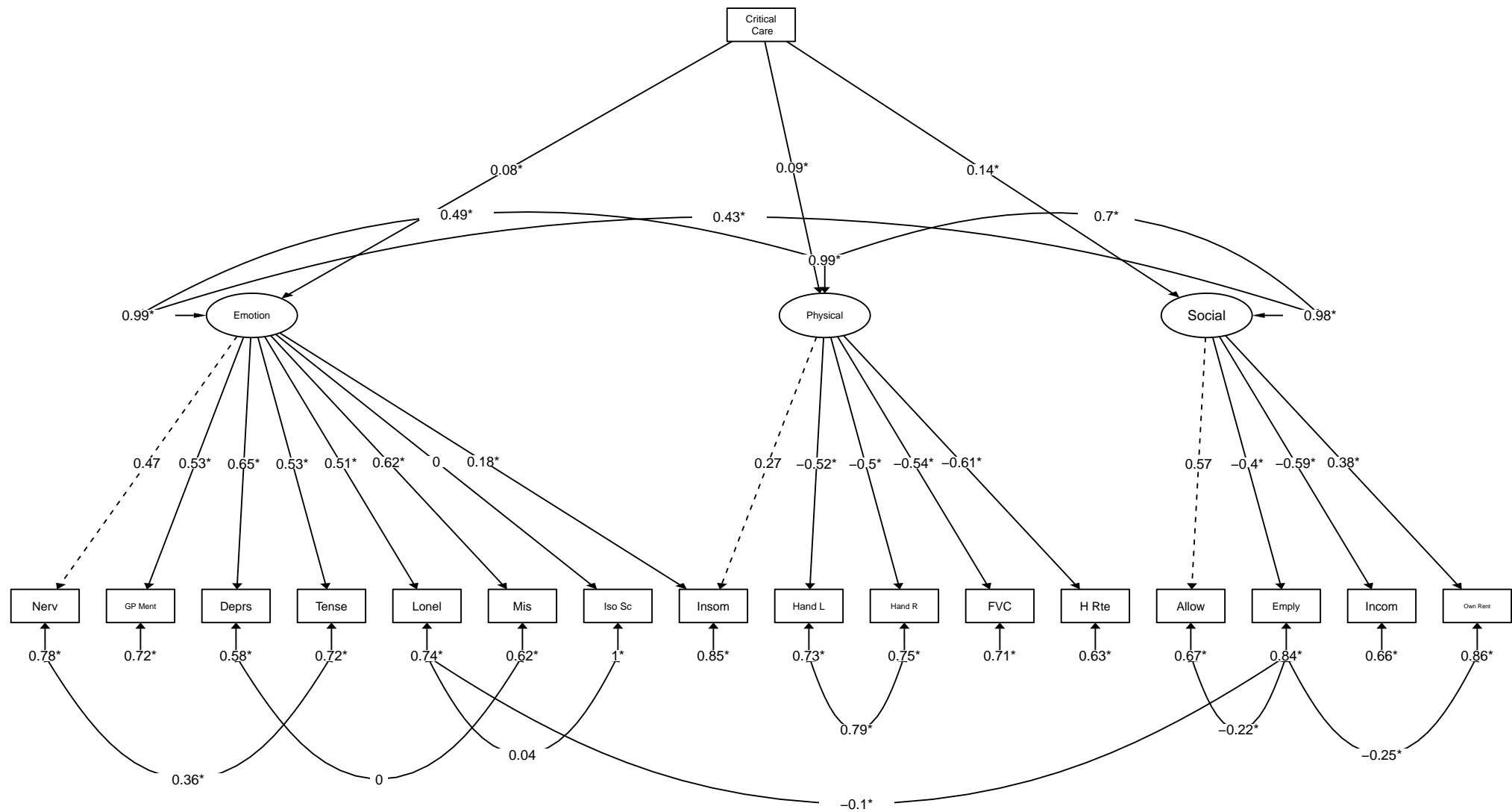

Supplement: Supplementary file 4 [file mmc4.pdf]

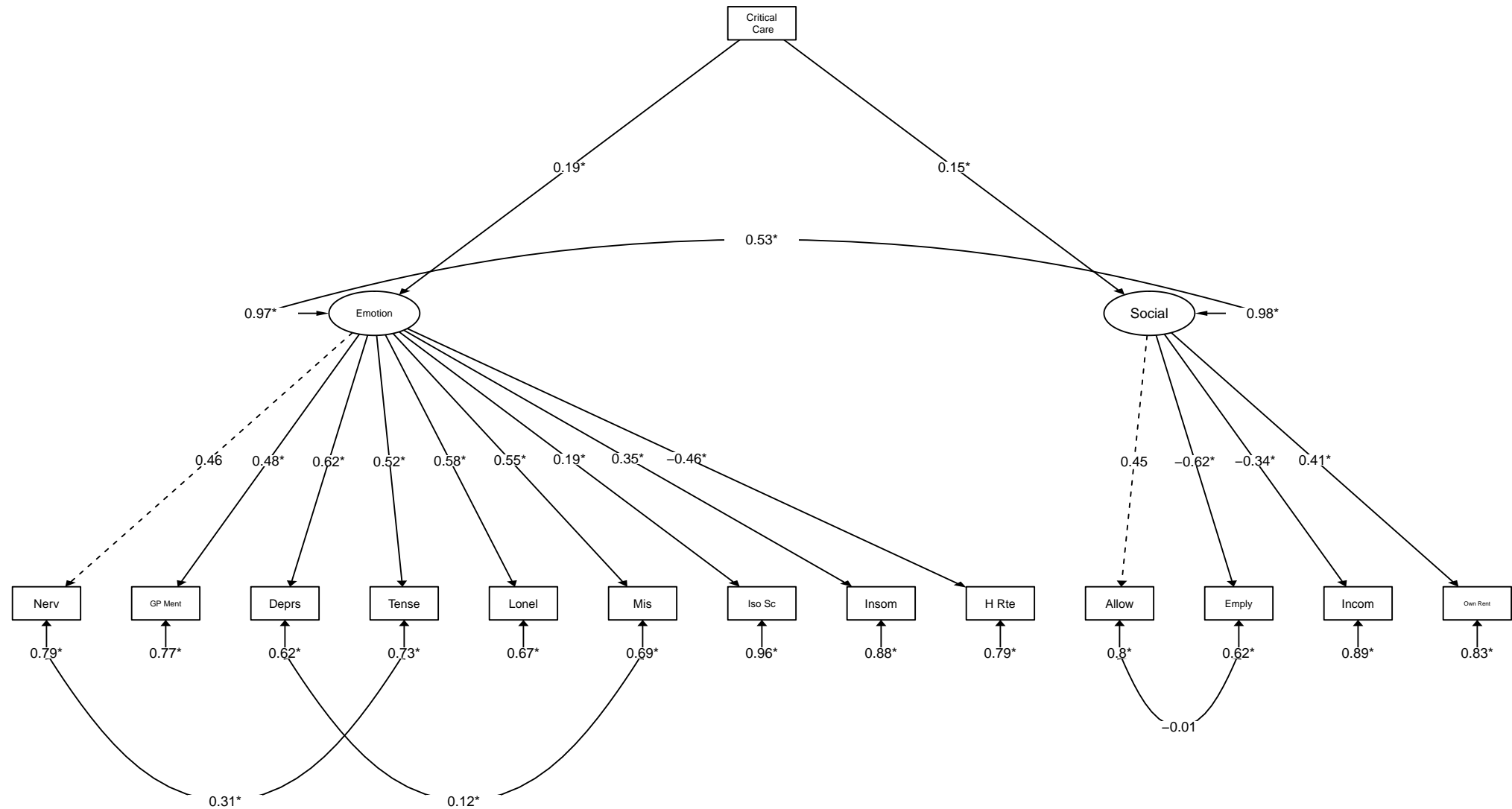

Supplement: Supplementary file 5 [file mmc5.pdf]
