# Supplementary material for: Long term outcomes following critical care hospital admission: A prospective cohort study of UK biobank participants
Source: Lancet Reg Health Eur. 2021 Jun 15;6:100121. doi: 10.1016/j.lanepe.2021.100121 (PMC8278491; doi:10.1016/j.lanepe.2021.100121)
Supplement: Supplementary file 3 [file mmc3.docx]

**S1: Supplementary File One: Variables and UK Biobank Identifiers used in this study**

| **Outcome** | **Biobank Identifier** |
| --- | --- |
| Overall Health Rating | 2178 |
| Frequency of depressed mood in the last two weeks | 2050 |
| Nervous feelings (Mental Health) | 1970 |
| Worrier/Anxious feelings | 1980 |
| Tense/highly strung | 1990 |
| Seen a GP for nerves, anxiety, tension or depression | 2090 |
| Sleeplessness/insomnia | 1200 |
| Miserableness | 1930 |
| Loneliness/isolation (Mental Health) | 2020 |
| Number in household | 709 |
| Leisure/social activities | 6160 |
| Frequency of friend/family visits | 1031 |
| Average household income before tax | 738 |
| Current employment status | 6142 |
| Own or rent accommodation lived in | 680 |
| Attendance/disability/mobility allowance | 6416 |
| Overall health rating | 2178 |
| Townsend deprivation index at recruitment | 189 |
| Ethnic Background | 21000 |
| Qualifications | 6138 |
| Consultant Speciality (Critical Care/ Intensive Therapy Unit) | 41246/41245 |
| Hand Grip Strength (left) | 46 |
| Hand Grip Strength (right) | 47 |
| Forced Vital Capacity | 3062 |
| MET minutes per week for all activity | 22040 |

*Household income (before tax)*

**Question asked**: What is the average total income before tax received by your household?

**Potential Answers**:

1. Less than £18,000
2. £18,000 to 30,999
3. £31,000 to £51,999
4. £52,000 to £100,000
5. Greater than £100,000
6. Do not know
7. Prefer not to answer

*Employment*

**Question asked:** Which of the following describes your current situation (you can select more than one answer)?

**Potential Answers:**

1. In paid employment or self employed
2. Retired
3. Looking after home/and or family
4. Unable to work because of sickness or disability
5. Unemployed
6. Doing unpaid or voluntary work
7. Full or part-time student
8. None of the above
9. Prefer not to answer

We grouped these responses into four categories:

**1)** Responses 1-3 *(Purposeful)*

**2)** Responses 4-5 (*Unable to work*)

**3)** Responses 6-7 (*Vocational Work*)

**4)** Responses 8-9 (*Not defined)*

*Government funded welfare support*

**Question asked:** Do you receive any of the following (you can select more than one answer)?

**Potential Answers:**

1. Attendance Allowance
2. Disability living allowance
3. Blue badge (Parking assistance)
4. None of the above
5. Do not know
6. Prefer not to answer

We classified Answers 1 and 2 as welfare support and compared the two study groups. Response three was analysed as a separate item.

*Housing Tenure*

**Question asked:** Do you own or rent the accommodation that you live in?

**Potential Answers:**

1. Own outright (by you or someone in your household)
2. Own with a mortgage
3. Rent- from local authority, local council, housing association
4. Rent- from private landlord or letting agency
5. Pay part rent and part mortgage (shared ownership)
6. Live in accommodation rent free
7. None of the above
8. Prefer not to answer

**Education Qualification explanation**

| **UK Definition** | **International relationship** |
| --- | --- |
| College/University Degree | College/University Degree |
| Other professional qualification | In job training leading to a professional qualification |
| A Levels/ AS Levels | Supplementary qualifications which allow direct access to University |
| NVQ/HND/HNC | Vocational qualification (i.e. electrician or trades person) |
| O Levels/GCSEs/CSEs | High School grade Graduation Diploma |

**S2: Supplementary File Two: Comorbidities assessed and utilised in matching**

Hypothyroidism

Uncomplicated Diabetes

Myocardial Infarction

Neurological Disorders

Renal Failure

Renal Disease

Deficiency Anaemia

Hypertension (with complications)

Solid tumour without metastasis

Malignancy (including lymphoma and leukaemia except malignant neoplasm of skin)

Chronic Pulmonary Disease

Rheumatoid Arthritis/Collagen Vascular Diseases

Rheumatic Disease

Valvular Disease

Alcohol Abuse

Peptic Ulcer Disease

Congestive Heart Failure

Cerebrovascular Disease

Hypertension (uncomplicated)

Obesity

Cardiac Arrhythmias

Peripheral Vascular Disorders

Liver Disease

Mild Liver Disease

Lymphoma

Pulmonary Circulation Disorders

Blood Loss Anaemia

Psychoses

Metastatic Cancer

Metastatic Solid Tumour

Diabetes Complicated

Coagulopathy

Fluid and electrolyte disorders

Moderate or severe liver disease

Paralysis

Hemiplegia or Paraplegia

Drug abuse

AIDS/HIV

Dementia

Weight Loss

Peptic Ulcer Disease (without bleeding complications)

Diabetes with chronic complication​

**S3: Supplementary File Three: Creation of the social isolation variable**

**Reference**

Morneau-Vaillancourt, G. Coleman, JRI. Purves, KL. Et al (2019) The genetic and environmental hierarchical structure of anxiety and depression in the UK Biobank. Depression and Anxiety; 37:512-520.

**S4: Matching Criteria and variables utilised**

Length of Hospital Stay

Emergency Admission

Surgical Admission

Admitting Diagnosis

Operation Type

Gender

Townsend Deprivation Index at Recruitment

Ethnicity

Education Qualifications

Smoking status

Time between hospital/critical care admission and assessment centre visit

Year of Admission

Age at hospital/critical care admission

Comorbidities (**see S2**).
